# Use of Iodide of Zinc

**Published:** 1856-04

**Authors:** D. L. M’Gugin

**Affiliations:** Prof. of Physiology, Pathology, &c.


### USE OF IODIDE OF ZINC.

BY D. L. M'GUGIN, M. D.,

Prof. of Physiology, Pathology, &c.

Several years since my attention was called to the use of the Iodide of Zinc in enlarged tonsils of a chronic character. A few cases soon presented themselves in which the tonsil instrument would have been otherwise the resort as the only means of relief. But by the use of the repeated application of a solution of ten grs. of the salt to ℥i of water, I have been able, in a large majority of cases, to overcome these chronic enlargements. Such were the fortunate results of the use of the remedy in these cases, that I was induced to try its efficacy in other cases.

I have since used it in ulceration of the Os Uteri. At first I used the solution and increased its strength. The result encouraged me in a perseverance in its use, and was decidedly better than usually follows the use of the nitrate of silver—in my hands at least. Instead of the solution, I now use the powder, by means of the hair pencil or a small piece of fine sponge on a probang and carried through the speculum to the ulcerated spot. A few applications, with some days interval, were all that have been necessary. The solution to the enlarged neck or lips of the uterus will be found a valuable local alterative.

To the ulcers of "nursing sore mouth," to the fauces in scarlatina, to foul ulcers upon the extremities, the solution has been very often followed by excellent results.

Time will not allow me now to give a report in detail of any one of the different cases in which I have used the Iodide of Zinc, but because I regard it, after considerable experience, as not only a valuable remedy, but preferable even to nitr. arg. in very many cases. I have thought proper in this brief article to call the attention of the profession to it and hope that after such a trial as I have given it, the result may be as satisfactory.

